# FGF2 Affects Parkinson’s Disease-Associated Molecular Networks Through Exosomal Rab8b/Rab31

**DOI:** 10.3389/fgene.2020.572058

**Published:** 2020-09-25

**Authors:** Rohit Kumar, Sainitin Donakonda, Stephan A. Müller, Kai Bötzel, Günter U. Höglinger, Thomas Koeglsperger

**Affiliations:** ^1^German Center for Neurodegenerative Diseases (DZNE), Munich, Germany; ^2^Faculty of Medicine, Klinikum Rechts der Isar, Technical University of Munich, Munich, Germany; ^3^Department of Neurology, Ludwig Maximilian University, Munich, Germany; ^4^Institute of Immunology and Experimental Oncology, Technical University of Munich, Munich, Germany; ^5^Department of Neurology, Hannover Medical School, Hanover, Germany

**Keywords:** Parkinson’s disease, exosomes, Rab proteins, membrane-trafficking, vesicular transport

## Abstract

Ras-associated binding (Rab) proteins are small GTPases that regulate the trafficking of membrane components during endocytosis and exocytosis including the release of extracellular vesicles (EVs). Parkinson’s disease (PD) is one of the most prevalent neurodegenerative disorder in the elderly population, where pathological proteins such as alpha-synuclein (α-Syn) are transmitted in EVs from one neuron to another neuron and ultimately across brain regions, thereby facilitating the spreading of pathology. We recently demonstrated fibroblast growth factor-2 (FGF2) to enhance the release of EVs and delineated the proteomic signature of FGF2-triggered EVs in cultured primary hippocampal neurons. Out of 235 significantly upregulated proteins, we found that FGF2 specifically enriched EVs for the two Rab family members *Rab8b* and *Rab31*. Consequently, we investigated the interactions of *Rab8b* and *Rab31* using a network analysis approach in order to estimate the global influence of their enrichment in EVs. To achieve this, we have demarcated a protein–protein interaction network (PPiN) for these Rabs and identified the proteins associated with PD in various cellular components of the central nervous system (CNS), in different brain regions, and in the enteric nervous system (ENS). A total of 126 direct or indirect interactions were reported for two Rab candidates, out of which 114 are *Rab8b* interactions and 54 are *Rab31* interactions, ultimately resulting in an individual interaction score (IS) of 90.48 and 42.86%, respectively. Conclusively, these results for the first time demonstrate the relevance of FGF2-induced Rab-enrichment in EVs and its potential to regulate PD pathophysiology.

## Introduction

Parkinson’s disease (PD) is a progressive neurodegenerative disorder characterized by the loss of neurons in substantia nigra pars compacta (SNc) and the gradual appearance of intraneuronal protein aggregates, termed Lewy bodies (LBs) (McCann et al., 2016). Neuropathological investigations in PD cases proposed that LBs first appear either in the olfactory bulb (OB) or the dorsal motor nucleus of the vagus (DMV) in the caudal medulla (Braak et al., 2003b). Prior to central nervous system (CNS) manifestation, Lewy pathology (LP) has therefore been assumed to begin in the gastro-intestinal tract as demonstrated by the presence of Lewy neurites (LNs) in the enteric nervous system (ENS) of PD patients (Holmqvist et al., 2014; Sanchez-Ferro et al., 2015). The gradual appearance of LBs in different brain regions correlates with progressive PD symptoms at distinct disease stages (Braak and Del Tredici, 2017).

Dementia in PD patients is associated to the appearance of LBs in hippocampal neurons together with a decrease in cholinergic transmission (Hall et al., 2014). LBs are largely composed of alpha-synuclein (α-Syn) and likely result from genetic factors and organelle dysfunction (Shults, 2006). Previous *in vitro* and *in vivo* studies demonstrate that α-Syn may be transferred from cell to cell, thereby promoting the “spread” of LP between different brain regions (Li et al., 2008; Cavaliere et al., 2017). It has been shown that α-Syn transfer can further seed the formation of LBs (Jones et al., 2015). Extracellular vesicles (EVs) from patients of dementia with LBs (DLB) enhanced the formation of α-Syn aggregates in the mouse brain, thus validating the idea of propagation via EV-mediated content exchange (Ngolab et al., 2017). It has been observed that EVs carry pathogenic α-Syn species to enhance the α-Syn aggregation process (Grey et al., 2015; Stuendl et al., 2016). Conversely, α-Syn is found capable of promoting EV release in CNS cells (Chang et al., 2013). The capacities of α-Syn to modulate vesicular populations are also known (Dettmer et al., 2017) and mutations of α-Syn were shown to defect the neuron’s endocytic machinery and impair vesicular transport in neurons, which is an earlier step of EV biogenesis pathway (Volpicelli-Daley et al., 2014; Xu et al., 2016). As a result, early-stage PD pathology exhibits a dysfunction in intracellular trafficking mechanisms (Hunn et al., 2015). Furthermore, it is reported that endoplasmic reticulum (ER)-Golgi vesicle trafficking genes, i.e., Ras-associated binding proteins (Rabs), modulate α-Syn-induced toxicity and share functional associations with α-Syn (Cooper et al., 2006; Breda et al., 2015). Rabs in general are one of the most abundant family of proteins and are involved in regulation of cellular functions such as intracellular component trafficking of organelles, proteins, and membranes via their binary mode of activation contingent to GTP/GDP bound states (Villarroel-Campos et al., 2016). In neurons, these Rab-mediated intracellular trafficking is associated to regulate the developmental processes (Shikanai et al., 2018). Processes such as neurodevelopment along with many others are governed via cross-communication among the nervous system (NS) components to which vesicular trafficking is at the center and largely regulated by Rab members (Ng and Tang, 2008). Furthermore, Rabs-mediated regulation of vesicular trafficking involves them to control the mechanisms of synaptic function, therefore making them worthy participants of both normal and disease pathophysiology altogether (Kiral et al., 2018; Mignogna and D’Adamo, 2018), though a comprehensive account of their roles in NS is unavailable. Continuous efforts have been made to classify and determine their functions and mode of actions (Le et al., 2018, 2019).

We recently demonstrated that fibroblast growth factor-2 (FGF2), a modulator of neuronal cholinergic activity, increases the release of EVs (Araujo et al., 1993; Kumar et al., 2020). In this recently published study, we performed mass spectrometry (MS) and found that FGF2 has significantly altered the EV proteome, leading to 441 proteins with a fold change of 1.5 and above. In total, 235 upregulated and 206 downregulated proteins were screened for members of the Rab protein family. We found that FGF2-triggered EVs were specifically enriched for *Rab8b* and *Rab31*. Hence, to further conclude on the global effects of *Rab8b* and *Rab31* enrichment and their importance in PD pathology, we performed protein–protein interaction network (PPiN) analysis and identified their interactions in different CNS-resident cell types, in different brain regions, and in ENS, highlighting the novel role of FGF2 in PD pathophysiology and paving a way forward to investigating the specific role of FGF2 in PD.

## Materials and Methods

### EV Proteome From Primary Hippocampal Neurons

All animal experiments were conducted in accordance with the regulations of the local animal welfare committee. Primary hippocampal neurons were cultured in a round Nunc culture dish (Thermo Fisher Scientific) from embryonic day 18 Charles River (CD) rats as described previously in Kumar et al. (2020). The EV pellets were harvested from the culture media of neurons treated with FGF2 (50 ng/ml) as per the guidelines provided by the International Society for Extracellular Vesicles (ISEV). The information on EV isolation method is submitted to EV TRACK (^1^ EV-TRACK ID: EV200085). The isolated EV pellets were taken forward to perform MS analysis as described in Kumar et al. (2020). The MS results of differential EV proteomic signature were then subjected to further bioinformatics analysis.

### Validation of *Rab8b* and *Rab31* Abundance

The abundant expression of *Rab8b* and *Rab31* in EV pellets was confirmed via Western blot technique as described in Kumar et al. (2020). In brief, blots were prepared using Mini-Protean Gels from Bio-Rad and Tris-glycine-based buffer. Polyvinylidene difluoride (PVDF) membranes (Bio−Rad) were used to blot the proteins at 150 mA for 60–120 min while keeping them on ice. 5% skimmed milk in TBST buffer (tris−buffered saline containing 0.05% tween) was used as blocking solution for 1 h. After washing, membranes were incubated with primary antibodies [Rabbit polyclonal Rab8B (# PA5-67354) and Rabbit polyclonal Rab31 (# PA5-54064) (Thermo Fisher Scientific)] overnight at 4°C. The next day, membranes were washed and incubated with the respective secondary antibody. The blots were then developed by using Clarity Western blot ECL Substrate (Bio−Rad). LI−COR Odyssey Fc Imaging system was used to detect the chemiluminescence and analyzed by Image Studio software (Licor). Western blot images were than used to calibrate the relative abundance of both candidate Rabs.

### PPiN, Pathways, and Cellular Component Enrichment Analysis

To construct a PPiN, we utilized the STRING v11 (accessed on 12/02/2020^2^) (Szklarczyk et al., 2019) online database. This resource assembles all known and predicted protein–protein interactions of organisms and consists of PPis based on the available experimental, text mining, and co-expression evidence. We retrieved the interactome (organism: *Rattus norvegicus*) of *Rab8b* and *Rab31* with the following criteria: (McCann et al., 2016) we considered an interaction as statistically significant at a >0.7 high confidence score (this cutoff is recommended by developers of STRING v11) (Braak et al., 2003b). To deduct all direct and indirect interactions of *Rab8b* and *Rab31*, we added the first shell of 100 and the second shell of 50 interactions. The *Rab8b* and *Rab31* interactome (RRi) map was visualized in Cytoscape v3.7.1 (Shannon et al., 2003). We calculated the average clustering coefficient and network heterogeneity using the Network Analyzer Cytoscape plugin (Shannon et al., 2003). The random network was generated based on the Erdos Renyi G (*n*,*p*) model using the Network Randomizer Cytoscape plugin (Shannon et al., 2003). The pathway annotation of RRi was performed using METASCAPE (accessed on 12/02/2020^3^) (Zhou et al., 2019), which contains the biological process (BP) annotations and cellular components mined from gene ontology and pathways from KEGG. We considered functions and cellular components as statistically significant using a *P* value cutoff of <0.05.

### Cell Type and Brain Region Enrichment Analysis

To determine which proteins from the RRi are enriched in different brain cell types (microglia, oligodendrocytes, astrocytes, and neurons) and regions (optic nerve, brain stem, cerebellum, corpus callosum, OB, striatum, hippocampus, motor cortex, prefrontal cortex, and thalamus), we downloaded the lists of these proteins from the following site^4^ (accessed on 14/02/2020) (Sharma et al., 2015). Prior to overlap analysis, we converted these mouse-specific gene symbols (we used gene symbols of proteome data) to rat ortholog gene symbols using the gProfiler online tool (^5^ accessed on 14/02/2020) (Raudvere et al., 2019). We cross-referenced the rat gene symbols with RRi and assessed the enrichment of brain cell types and region proteins in RRi.

### ENS Analysis

To deduce the connection between the ENS and the RRi, we extracted the transcriptome signature from Roy-Carson et al. (2017). In this study, the authors generated gene expression profiles of a lineage-specific population of enteric progenitors in zebrafish. We collected the up- and downregulated genes produced in Roy-Carson et al. (2017). We converted zebrafish gene symbols to rat orthologs (gene symbols) using the gProfiler online tool (see text footnote 5, accessed on 15/02/2020) (Raudvere et al., 2019). We mapped rat orthologs obtained from gProfiler onto RRi using Cytoscape v3.7.1 (Shannon et al., 2003).

### Mining of PD Proteins

To define if any Parkinson’s disease (PD)-related proteins are enriched in RRis, we extracted PD proteome screens from publications (Dumitriu et al., 2016; Boerger et al., 2019; Lachen-Montes et al., 2019). Furthermore, we obtained PD-associated genes from the following resources: DisGeNET (^6^ accessed on 16/02/2020) and Rat Genome Database (RGD) (^7^ accessed on: 16/02/2020). We mined PD-associated genes from all the sources as mentioned above and translated their gene symbols to rat gene symbols using the gProfiler tool (accessed on 16/02/2020) (Raudvere et al., 2019). This list of rat gene symbols related to PD was finally intersected with RRi.

### Sequence Retrieval and Structural Modeling

To model the structures of Rab8b, Rab3b, and Rab23 from the rat organism, we first retrieved protein sequences in fasta format from UniProt online database (^8^ accessed on 22/07/2020). Rab8b (UniProt ID: P70550), Rab3b (UniProt ID: Q63941), and Rab23 (UniProt ID: D3ZRM5) sequences were used as an input to SWISS-MODEL (Waterhouse et al., 2018) online software (^9^ accessed on 22/07/2020) to model the three-dimensional (3D) protein structures. Rab8b was modeled based on template from Protein Data Bank (PDB) database (PDB ID: 6rlr.1.B); Rab23 and Rab3b templates were also obtained from PDB (PDB IDs: 1z2a.1.A and 3dz8.1.A, respectively). The 3D models were selected for further analysis based on the Global Model Quality Estimation (GQME) and Qualitative Model Energy Analysis (QMEAN) values. GQME values range from 0 to 1; close to 1 represents reliability of the predicted 3D structure and QMEAN value <4.0 shows trustworthiness of structure. Then, atomic details such as correcting the side chains of structures were determined using the ModRefiner online tool (^10^ accessed on 22/07/2020) (Xu and Zhang, 2011), and the Chiron online tool (^11^ accessed on 22/07/2020) (Ramachandran et al., 2011) was used to remove steric clashes. ProSA-web online algorithm (Wiederstein and Sippl, 2007) (^12^ accessed on 22/07/2020) was used to calculate the *z* score of the 3D structures to understand how well it is compared to experimental structures derived from X-ray or NMR.

### Validation of Protein–Protein Interaction Using Docking

To predict the interaction between the Rab8b–Rab3b and Rab8b–Rab23 at the sequence level, we utilized protein sequences obtained from UniProt online database (see text footnote 8, accessed on 23/07/2020) and used the BIPSPI online tool (^13^ accessed on 23/07/2020) (Sanchez-Garcia et al., 2019) to compute the interaction between the proteins. Then, we performed protein–protein (Rab8b–Rab3b) and (Rab8b–Rab23) docking to understand these interactions using a hybrid docking strategy with the HDOCK online server (^14^ accessed on 23/07/2020) (Yan et al., 2017). Rab8b was used as receptor, and Rab3b and Rab23 were used as ligands. We used predicted binding site residues from sequence level analysis from the BIPSPI tool during docking analysis and default parameters. The docking scores between the protein–protein were computed using improved shape-based, pairwise scoring function in HDOCK.

### Statistical Evaluation and Data Visualization

The statistical evaluation and visualization in this study, if not stated otherwise, was executed using the R statistical environment v3.5.1^15^. Scatter plot, boxplot, and barplot were visualized using the ggplot2 R package. The PD-enriched protein heatmap was generated using Pheatmap R package. The percentage of *Rab8b* and *Rab31* direct and indirect interactions is visualized as a donut plot in Microsoft Excel. Statistical assessment of cell type and brain region protein overlap with RRi was done using Fisher’s exact test, multiple comparisons were adjusted by the false discovery rate (FDR) (*P* < 0.05) Benjamini–Hochberg (BH) method, and *P* values were converted to negative log_10_. 3D protein structure pictures were rendered in PyMOL software v2.3.0.

## Results

### FGF2 Enriches *Rab8b* and *Rab31* in Neuronal EVs

**FIGURE 1:**
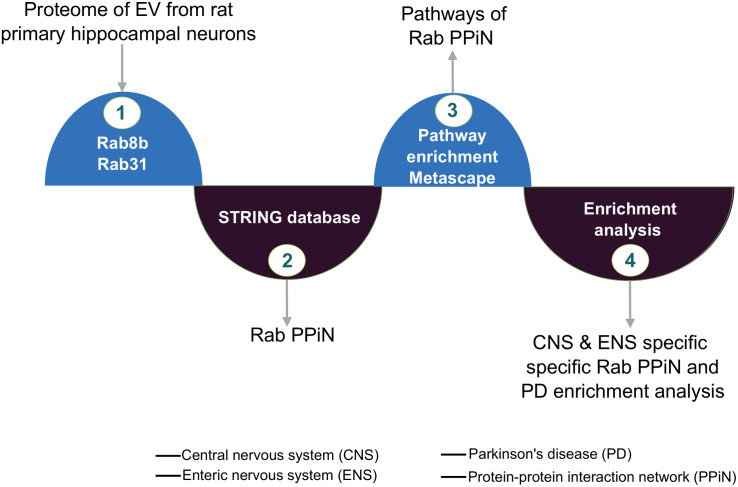
FGF2-mediated Rab protein enrichment and interactions. Graphic illustrating *Rab8b* and *Rab31* protein’s network and the integrated analysis from extracellular vesicle (EV) proteome.

Using at least three biological replicates, we recently assessed the global proteomic changes in EVs in response to treatment with FGF2 by performing a thorough MS analysis (Kumar et al., 2020). Out of 2258 differentially expressed proteins (DEPs), which were detected at least once in all samples, 705 were significantly expressed (FDR ≤ 0.05) and 441 proteins had a fold change of 1.5 or above (Kumar et al., 2020). Among the upregulated candidates, we found *Rab8b* and *Rab31* as prominent members of the Rab protein family with an increased abundance in EV pellets (Supplementary Figure 1). We cross-validated the proportional expression of these candidates along with EV biomarkers from their expression signals in MS data (Supplementary Figure 2). This enrichment was then considered for further analysis by subjecting it to a bioinformatic analysis (Figure 1).

### *Rab8b* Is a Key Effector in the *Rab8b*–*Rab31* Interactome

**FIGURE 2:**
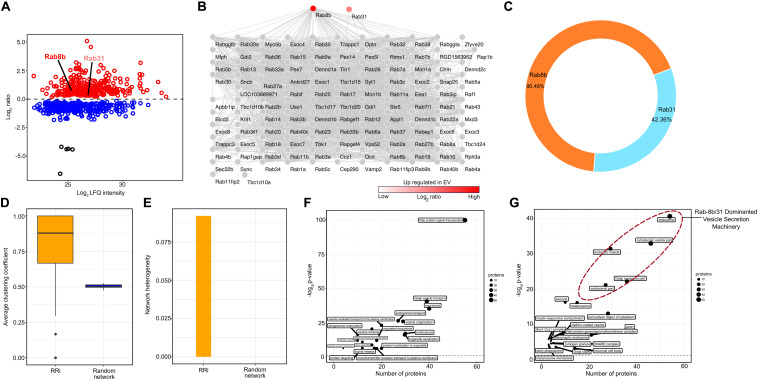
Protein–protein network and pathway analysis of Rab8b and Rab31 proteins. **(A)** Scatter plot showing enriched proteins in EV in response to treatment with FGF2. Two Rab proteins *Rab8b* and *Rab31* are upregulated (red color) in bFGF-induced EV proteome. **(B)** Network representation RRi derived from STRING DB. **(C)** Donut plot showing the percentages of direct and indirect interactions of *Rab8b* and *Rab3*. **(D)** Average clustering coefficient distributions for the RRi and random networks. **(E)** Network heterogeneity of RRi and random networks. **(F,G)** Scatter plot of enriched biological processes and KEGG pathways and cellular components of RRi. The –log_10_ *P* value indicates enrichment of pathway. Note: red color indicates upregulation.

The EV proteome analysis revealed that FGF2 affects two specific Rab proteins, i.e., *Rab8b* and *Rab31*, where FGF2 increased the abundance of *Rab8b* up to 2-fold and that of *Rab31* up to ∼1.5-fold (Figure 2A). In order to visualize the interactive network of these Rabs, we performed a PPiN analysis to assess the full potential of FGF2-prompted *Rab8b* and *Rab31*. We extracted direct and indirect interaction partners through STRINGDB, yielding a *Rab8b*–*Rab31* interactome (RRi) (Figure 2B). At a very high confidence threshold cutoff (>0.7), a total of 126 *Rab8b* and *Rab31* interactions were found. Among overlapping interacting proteins, a total number of 114 and 54 interactions were attributable to *Rab8b* and *Rab31*, respectively. This resulted in an individual interaction percentage of 90.48% for *Rab8b* and 42.86% for *Rab31*, making *Rab8b* the top contributor in the RRi (Figure 2C). Next, we examined the key topological properties such as average clustering coefficient (avg.cc) and network heterogeneity (nhet). We first computed the avg.cc in the RRi, compared it to a random network, and found significantly higher avg.cc in the RRi (Figure 2D). We observed higher nhet values for the RRi in contrast to a random network (Figure 2E). Taken together, our RRi analysis revealed *Rab8b* as a key effector. Furthermore, topological analysis showed that RRi is modular and very heterogeneous in nature, thus confirming that the interactome is controlled by central proteins.

### Neuronal Vesicle Release Sites Are Functional Epicenters of RRi

We obtained a functional readout of the RRi by subjecting the RRi dignitaries to a pathway enrichment analysis and found a number of vesicular secretion-related pathways enriched (Figure 2F). A higher significance and a high number of ∼50 candidates enriching Rab protein signal transduction pathway was apparent from the participants of RRi. Pathways involved in vesicle secretion and membrane trafficking, e.g., Golgi vesicle transport, exocytosis, endocytosis, and protein localization to organelle, had a participation of ∼40 candidates, each scoring a very high significant *P* value cutoff of <0.05. Approximately 30 RRi members each exhibited a direct input to neuronal transport pathways like endosomal transport, vesicle-mediated transport to the plasma membrane, vesicle docking, protein localization to organelles, and neurotransmitter receptor transport to the plasma membrane. Other pathways such as phagosome maturation, endocytic recycling, signal release, and protein targeting were contributed by ∼20 nominees of each RRi and were significantly enriched. A detailed information on pathway participation by each candidate is included in Supplementary Table 1. The functional work stations of the RRi were attributed to the major neuronal compartments governing vesicle secretion (Figure 2G). Nearly 50 proteins were designated to an endosomal subcellular location, ∼40 to cytoplasmic vesicles, and ∼30 each to endocytic vesicles, the Golgi apparatus, and perinuclear regions of the cytoplasm including the major secretory machinery of neurons. Other subcellular locations involved in secretory processes such as post-synaptic endosomes, SNARE complexes, neuronal cell body, clathrin-coated vesicles, and early phagosome were recognized with a participatory candidature of ∼ 20 and 10 proteins each and were found above the significance cutoff. The individual candidature of proteins to subcellular locations is reported in Supplementary Table 2. Conclusively, from the subcellular location of RRi members, we concluded that FGF2-triggered enrichment of *Rab8b* and *Rab31* to EVs will likely influence early steps of EV biogenesis.

### RRi Has Its Implications to Other Native Brain Regions and Cells

**FIGURE 3:**
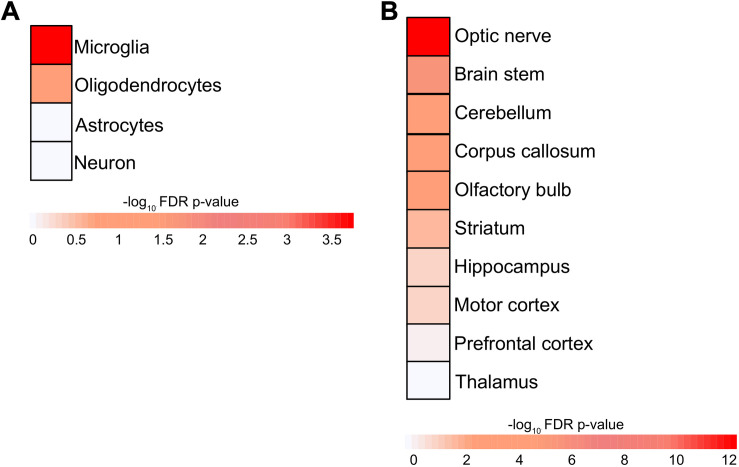
Enrichment of brain cell types and region in RRi. **(A)** Heatmap representing the microglia, oligodendrocyte, astrocyte, neuron cell type enrichment. **(B)** Heatmap showing the enrichment of optic nerve, brain stem, cerebellum, corpus callosum, olfactory bulb, striatum, hippocampus, motor cortex, prefrontal cortex, and thalamus brain regions. The enrichments were assessed using Fisher’s exact test and negative log_10_ Benjamini–Hochberg-adjusted *P* values; statistical significance cutoff was set at 1.31 and is comparable to the *P* values ≤ 0.05.

Extracellular vesicles are capable of transmitting biomaterials from one cell to another and between different brain regions and are released by all CNS-resident cell types; therefore, an overlapping molecular machinery is expected. In order to investigate the potential interactions of RRis in other brain cells, regions, and their peripheral extensions, we have mapped RRi proteins (RRiPs) to indigenous proteomic signatures of respective neuronal components, cell types, and regions. This resulted in the highest enrichment (*P* < 0.05) for glial cells, i.e., microglia and oligodendrocytes (Figure 3A). In line, our analysis depicted a greater overlap for the optic nerve, brain stem, cerebellum, corpus callosum, OB, and striatum native RRiPs (Figure 3B). The results thus highlight the possible interaction of FGF2-stimulated EV-*Rab8b* and *Rab31* to the aforementioned respective sites.

### Annotation of Neuroglial Rabs Delineates the RRi Roles in Glial Action

**FIGURE 4:**
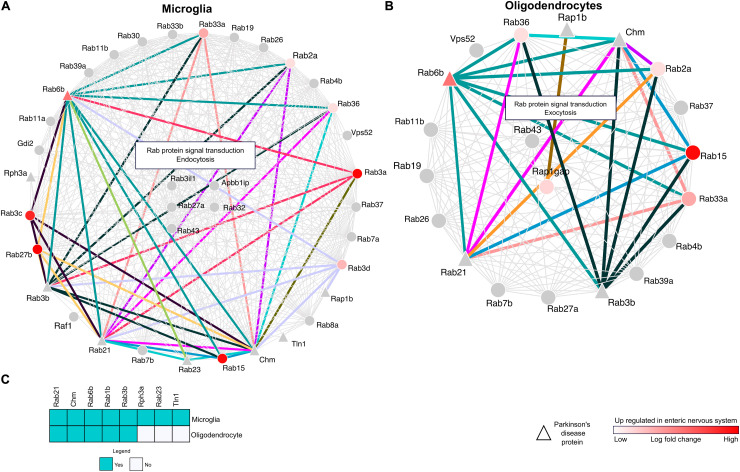
Cell-type-specific and enteric nervous system (ENS) protein interactions. **(A,B)** Network maps representing the RRi and pathways regulated in microglia and oligodendrocytes. Note: red color denotes upregulation of a protein in the ENS. Triangle shape nodes indicate Parkinson’s disease (PD)-related proteins. Colored lines denote the interactions between the PD and ENS proteins. **(C)** The heatmap represents the PD proteins between the microglia and oligodendrocytes.

To comprehend the neuroglial significance of RRi, we considered the hub Rab proteins reflected in significantly enriched glial components and developed a microglial specific Rab interactome (mSRi) (Figure 4A) and oligodendrocyte-specific Rab interactome (oSRi) (Figure 4B). The mSRi had *Rab3il1*, *Rab27a*, *Rab32*, *Rab43*, and *Apbb1ip* as hub Rabs, whereas *Rab43* and *Rap1gap* were the hub proteins of oSRi. We individually subjected mSRi and oSRi to pathway enrichment analysis and found Rab protein signal transduction, endocytosis, and exocytosis as the top significantly enriched pathways. Interestingly, we observed an alternative enrichment of biological pathways for the mSRi and oSRi, with endocytosis as top hit of mSRi-Rabs and exocytosis of oSRi-Rabs allowing one to venture the possible global molecular interactions as a result of high FGF2 level in neurons.

### Pathological Relevance of mSRi and oSRi Along the Gut–Brain Axis Is Established by PD-ENS Rabs

As introduced earlier, the possibilities imposed by LB formation and its appearance in various CNS–ENS components and Rab interactions from mSRi and oSRi were identified in the background of their weight to the ENS, and their further ENS-PD participation was determined. We identified 71 ENS-PD-specific interactions, with 46 in mSRi and 25 in oSRi (Figures 4A,B). In mSRi, we identified *Rab6b*, *Rab3b*, *Rab21*, *Chm*, and *Rab23* as PD-Rabs, which had shown interactions with ENS-Rabs. *Rab6b* interacts with 10 other Rabs (*Rab2a*, *Rab3a*, *Rab3b*, *Rab3c*, *Rab3d*, *Rab33a*, *Rab15*, *Rab21*, *Rab23*, and *Rab27b*), which are present in ENS. Similarly, PD-Rabs *Rab3b* and *Chm* have 10 and 12 interactions with ENS-Rabs, respectively. PD-Rab *Rab23* and *Rab21* were found to be interacting with 3 and 11 other ENS-Rabs, respectively. Out of the total 25 PD-ENS oSRi Rab interactions, *Rab6b*, *Rab3b*, *Rab21*, and *Chm* had six interactions each; PD-Rab *Rap1b* had shown to be interacting with one *Rap1gap* ENS-Rab. All interaction nodes specific to PD-ENS Rabs were shown conjoined by colored lines between the nodes (Figures 4A,B) and the individual functional contributions of PD-ENS interactions are presented in Supplementary Table 3. PD-Rabs *Rab2*, *Chm*, *Rab6b*, *Rab1b*, and *Rab3b* have shared candidature to both mSRi and oSRi, though *Rph3a*, *Rab23*, and *Tln1* were microglia-specific PD-associated Rabs (Figure 4C).

### Alternative Functional Enrichment by Brain-Region-Specific Rabs Sketches the FGF2-Induced EV-Mediated Pathology Offshoot

**FIGURE 5:**
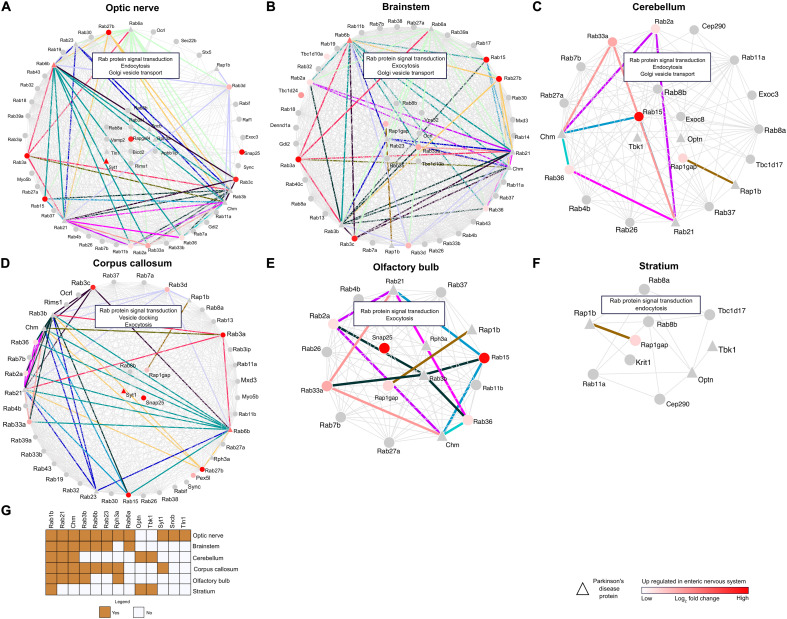
Network analysis of brain region- and ENS-specific proteins. **(A–F)** Network plots showing the RRi and pathways regulated in the optic nerve, brain stem, cerebellum, corpus callosum, olfactory bulb, and striatum. Note: red color indicates upregulation of a protein in the ENS. Triangle shape nodes denote PD-related proteins. Colored lines indicate the interactions between the PD and ENS proteins. **(G)** The heatmap signifies the PD proteins between the brain regions.

The RRiPs were mapped to CNS-specific proteome signatures, and significantly enriched regions were considered for further analyses of region-specific Rab interactions. Brain-region-specific sub-RRis for each significantly enriched region were developed. This approach resulted in optic-nerve-specific Rab infraction (onSRi) (Figure 5A), brain-stem-specific Rab infraction (bsSRi) (Figure 5B), cerebellum-specific Rab infraction (ceSRi) (Figure 5C), corpus-callosum-specific Rab infraction (ccSRi) (Figure 5D), olfactory-bulb-specific Rab infraction (obSRi) (Figure 5E), and striatum-specific Rab infraction (stSRi) (Figure 5F) subnets. The functional hubs of these interactions in sub-RRis were detected, and these hub Rab proteins would therefore represent the key Rab molecules in the respective anatomical brain segment under the potential influence of FGF2 and may therefore govern the CNS-specific Rab infractions. For onSRi, *Rab8b*, *Rab8a*, *Rab3il1*, *Sncb*, *Rph3a*, *Vamp2*, *Rapgef4*, *Tln1*, *Bicd2*, *Apbb1ip*, *Rims1*, and *Syt1* were the hub proteins along with Rab signal transduction, endocytosis, and Golgi vesicle transport as the key enriched biological pathways. In bsSRi, we found *Rab8b*, *Vps52*, *Rap1gap*, *Ocrl*, *Rab23*, *Rab33a*, *Rab35*, and *Tbc1d10b* as key hubs contributing to biological pathway Rab signal transduction, exocytosis, and Golgi vesicle transport. The ceSRi hubs were *Rab8b*, *Exoc8*, *Optn*, *Rab15*, and *Tbk1* enriching Rab signal transduction, endocytosis, and Golgi vesicle transport as top functional pathways via their interactions. *Rab8b*, *Rap1gap*, *Syt1*, and *Snap25* were the hub molecules in ccSRi showing top enrichment of Rab signal transduction, exocytosis, and vesicle docking pathways. For obSRi, key governing molecules were *Snap25*, *Rph3A*, *Rap1gap*, and *Rab3b* sharing Rab signal transduction and exocytosis as top enriched pathways altogether from their interactions. *Rab8b* and *Rap1gap* were the central molecules for stSRi and enriched Rab signal transduction and endocytosis as main pathways. The enrichment of Rab signal transduction as the key pathway among all sub-RRis is obvious from the key Rab interactions, also highlighting the alternative enrichment of exocytosis and endocytosis as core functional enrichment to various brain regions.

### Brain-Region-Specific PD-ENS Rabs Settle FGF2-Induced Rab Enrichment Along the Gut–Brain Axis

In total, 181 ENS-PD-specific interactions were identified among CNS-specific Rab infractions (CNS-RRi) in rat orthologs, out of which 46 were found in onSRi, 58 in bsSRi, 10 in ceSRi, 48 in ccSRi, 18 in obSRi, and 1 in stSRi (Figures 5A–F). Next, we performed functional enrichment analysis for PD-ENS interaction partners; the significantly enriched pathways are shown in Supplementary Table 4. Notably, we found the novel *Rab3b* interaction-mediated enrichment of the peptidyl-cysteine methylation pathway among all other PD-ENS interactions, where *Rab3b* is a common protein interacting with other Rabs and is native to the optic nerve and brain stem, emphasizing the epigenetic potentials of Rab interactions in PD pathology as methylation is one of the deriving reactions for epigenetic changes. This finding allows us to envision the optic nerve and, in particular, the brain stem as methylation-prone epicenters in PD pathophysiology. Interestingly, all PD-ENS interactions had enriched exocytosis-related biological pathways, whereas non-PD-ENS Rabs were selectively enriched with endocytosis-related pathways. Such selective alterations to pathway enrichment support the role of EV-mediated transport of pathological proteins in PD, therefore ultimately strengthening the overall zest of studying FGF2-enriched EV-Rabs and their potential for PD pathology. We found that a large number of PD-Rabs were native to onSRi and *Rab1b* was found in all CNS-RRi subnets (Figure 5G). PD-related *Optn* and *Tbk1* were only shared by ceSRi and stSRi. PD-related *Syt1* participated in onSRi and ccSRi subnets only.

### Rab8b Shows Strong Interactions With Native PD-Related Rab3b and Rab23

**FIGURE 6:**
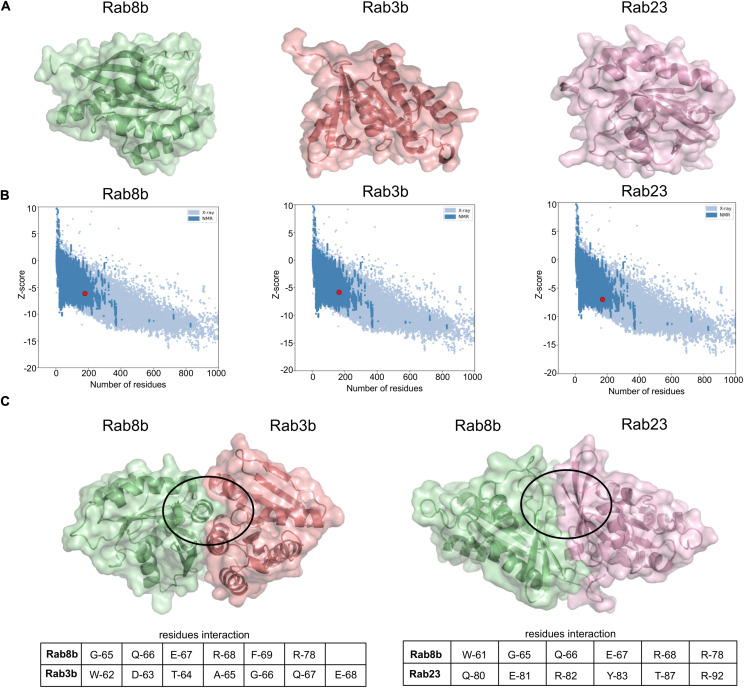
Interaction between *Rab8b* and their targets. **(A)** The 3D models of *Rab8b*, *Rab3b*, and *Rab23* modeled using SWISS-MODEL. **(B)** 3D models structure validation by ProSA. This plot represents modeled structure score, *Rab8b* (*z* score: –6.11), *Rab3b* (*z* score: –5.71), and *Rab23* (*z* score: –6.99) specified by the red dot, shows and that it is in the range of scores found on comparable sized proteins with an NMR quality. **(C)** The 3D structures *Rab8b–Rab3b* and *Rab8b–Rab23* interactions were built on the 3D models from SWISS-MODEL and docking simulation was done using HDOCK program. The circles highlight regions of interacting residues in the protein 3D models.

To computationally model interactions between Rab8b–Rab3b and Rab8b–Rab23, we used a two-layered approach. We first performed interaction analysis at sequence level for this purpose, and we utilized the BIPSPI method and predicted the residue–residue contacts between the proteins at the interface. This analysis revealed a Rab8b–Rab3b and Rab8b–Ra3b interaction score of 1.64 and 1.63, respectively (Supplementary Figure 3). Secondly, to understand these interactions at the structural level, we looked for 3D protein structures, but there were no experimentally derived protein structures related to Rab8b, Rab23, and Rab3b from the rat organism in the PDB. Thus, we computationally modeled the 3D protein structures of Rab8b, Rab3b, and Rab23 using SWISSMODEL (Figure 6A). The preliminary analysis of 3D models showed very good Global Model Quality Estimation (GQME) and Qualitative Model Energy Analysis (QMEAN) values for Rab8b (GQME: 0.79, QMEAN: −0.20), Rab8b (GQME: 0.76, QMEAN: −0.03), and Rab23 (GQME: 0.69, QMEAN: −0.43). Furthermore, we adjusted our predicted models at the atomic level and removed steric clashes. These analyses improved our predicted 3D models, and to further confirm this, we performed ProSA analysis (see *Materials and methods*) by comparing the 3D structures of Rab8b, Rab3b, and Rab23 with experimental structures obtained via X-ray and NMR methods. This analysis indicated that all Rab8b, Rab23, and Rab3b models are in the range of NMR-derived structures (*z* scores: −6.11, −5.71, and −6.99, respectively) (Figure 6B).

We validated the interactions between Rab8b–Rab3b at the structure level by performing docking analysis using the HDOCK server. The results included the following residues G-65, Q-66, E-67, R-68, F-69, and R-78 of Rab8b interacting with Rab3b residues W-62, D-63, T-64, A-65, 66-G, Q-67, and E-68 in the lowest docked energy model (−236.42) in contrast to 10 predicted docked complexes (Figure 6C and Supplementary Table 5). The docking between Rab8b–Rab23 revealed that W-61, G-65, Q-66, E-67, R-68, and R-78 residues of Rab8b interact with Q-80, E-81, R-82, Y-83, T-87, and R-92 residues of Rab23 with the lowest docked energy (−441.14) in comparison to 10 predicted docked complexes (Figure 6C and Supplementary Table 5). Taken together, the sequence and modeling analysis allowed us to predict the key interacting residues between Rab8b–Rab3b and Rab8b–Rab23, therefore validating these interactions.

## Discussion

Early onset of PD pathology is characterized by defects in intracellular trafficking mechanisms (Hunn et al., 2015). These trafficking mechanisms are predominantly regulated via the involvement of members of the Rab family. In our proteomic analysis, we observed a *Rab8b* and *Rab31* enrichment in EVs after FGF2 treatment of hippocampal neurons and, therefore, applied network and functional analysis methods to investigate the interactome of Rabs and their possible association to PD pathology having EV *Rab8b* and *Rab31* as guiding forerunners (Figures 1, 2A). The participation percentage from direct–indirect interactions of both Rabs in the *Rab8b*–*Rab31* interactome (RRi) (Figure 2B) established *Rab8b* as a key regulator of the RRi (Figure 2C). Topological analysis demonstrated that RRi is modular and consists of highly connected proteins (Figures 2D,E). Earlier studies confirmed that *Rab8b* is an interacting partner of Otoferlin, which is a protein associated to hearing loss (Heidrych et al., 2008). Several genetic alterations to Otoferlin are found to be associated to the pathogenicity of hearing-related disorders (Varga et al., 2006), and PD patients are found prone to hearing loss or having difficulties in processing auditory inputs (Folmer et al., 2017). Therefore, *Rab8b* enrichment in EVs stimulated by high FGF2 levels (reported for the first time in our study) and its interactions supports a novel possibility of explaining molecular basis of non-motor symptoms like hearing loss in PD pathology (Vitale et al., 2012). Furthermore, hearing loss is associated to dementia as reported in Lin et al. (2011), and the relevance of the hippocampus in DLB is known (Liu et al., 2019). Autopsies of PD and DLB patients suggests a spreading of pathology among the brain regions, a role of hippocampal α-Syn in the loss of cognitive functions, and additional non-motor symptoms across the parkinsonian pathology spectrum (Adamowicz et al., 2017). These results thus highlight the overall significance of our study on hippocampal neurons. The functional enrichment analysis and subcellular segregation of RRi members support the idea of FGF2 influencing EV biogenesis (Figures 2F,G). From earlier studies, it has been reported that *Rab8b* promotes the caveolin-mediated endocytosis of certain receptors during early endosome formation that subsequently fuse with multi-vesicular bodies (MVBs) and therefore facilitates the EV biogenesis (Demir et al., 2013). Moreover, *Rab31* has been shown to have a role in the endocytic trafficking of growth factor receptors and regulation of the early endosome antigen 1 (EEA1), a trafficking complex formation, which is a pre-step implicated in EV biogenesis (Chua et al., 2014). These early involvements in vesicle secretion cycle thus establish the roles of *Rab8b* and *Rab31* in the cargo-dependent fate of EVs, which is also counter supported by the fact that they are enriched to EVs specifically after FGF2 treatment.

The relevance of EV-associated Rab enrichment for PD pathology was further assessed via the global effect of RRi interactions, obtained from mapping RRi members to native proteomic orthologs, and we identified significantly enriched Rabs specific to CNS cells and anatomical regions (Figures 3A,B). The significant enrichment of glial cells in our mapping analysis may be due to the high availability of publicly accessible data, which are often derived from glial cells in the context of EVs. In addition, the fact that a high number of investigations focused mainly on secretory pathways may limit the conclusions of this work. Yet, the identification of an alternative functional enrichment by mSRi and oSRi Rabs supports previewing a Rab-mediated CNS homeostatic regulation sieged by high FGF2 levels and is likely primed via EV secretion (Figures 4A,B). The specific enrichment of exocytosis from oSRi and endocytosis by mSRi as key pathways supports a cross-talk concomitant to glial cells. The parallel appearance of LBs in glial cells further validates such cross-talk (Braak et al., 2007). It has been shown that inclusion burden in glial cells is directly proportional to the loss of SNc neurons (Wakabayashi et al., 2000). The appearance of *Apbb1ip* as one of the hub proteins in the Rab interactome and the presence of microglia-specific *Tln* in mSRi support the possibility of a FGF2-enhanced LB formation (Figures 4A,C). It has been reported that *Apbb1ip* directly interacts with *Tln* and is further required to activate integrins, a family of adhesion molecules (Lee et al., 2009). In leukocytes, it has been shown that a lack of *Apbb1ip* results in adhesion deficiency of leukocytes (Klapproth et al., 2015). Such adhesion deficit insinuations allow us to deduce a hypothetical model of LB formation by means of protein misfolding tethered through “protein-stickiness” and subsequently accompanied by other associated interactions of PD-related Rabs at high FGF2 levels. Furthermore, the hippocampal expression of *Apbb1ip* makes it a favorable candidate to discern the molecular pathophysiology of dementia in PD patients (Moradifard et al., 2018).

It became clear now that dopamine (DA) depletion does not correlate significantly with non-motor symptoms in PD patients and that they retain high growth factor levels with a distinct inflammatory profile (Brockmann et al., 2017; Park et al., 2019). Alongside the consecutive appearance of LBs, it is therefore important to consider cells and brain regions beyond SNc-DA neurons and examine the respective pathology correlates. Our mapping analysis allowed us to identify significantly enriched brain regions for RRi members and to develop a brain-region-specific Rab interactome (Figures 5A–F). Furthermore, alterations of the endocytosis/exocytosis pathway enrichment in different brain regions may explain how EVs transfer malicious proteins and ultimately enhance the pathology spread with Rab mediation. In line with exocytosis as the main pathway enriched in the brain stem, OB and corpus callosum could provide a preliminary map of “release centers,” whereas the alternative enrichment of endocytosis as the main pathway in other brain regions could act as “receiving centers.”

The appearance of *Ocrl* as a hub molecule in brain-stem-specific bsSRi (Figure 5B) is interesting, since the brain stem may act as an input point in a pathology progression interceded from the ENS to CNS (Braak et al., 2003a; Abbott et al., 2007). Therefore, we have resolved the ENS-allied Rabs in all the interactomes, which could possibly delineate the Rab-governed pathology along the gut–brain axis (Figures 4, 5). This could further be affirmed by the fact that *Ocrl* is involved in the regulation of early endosomes in a Rab-dependent manner and shares a binding site with *Appl1*, another endocytic protein (Schenck et al., 2008; Swan et al., 2010). The fate of such interactions could therefore further mediate the transmission of LB-associated content in EVs and hence establish the brain stem as a “relay center.” This gains further support by the finding that bsSRi candidates specifically designate exocytosis as the top outcome in our functional pathway enrichment analysis. The association of *Ocrl* to other disorders like Lowe syndrome and Dent disease signifies the role of these interactions beyond PD pathology and may serve as a merging point for many symptoms across the parkinsonian symptom spectrum (De Matteis et al., 2017). The presence of *Rab3b* among the top coinciding PD-Rabs (Figure 5G) may explain a possible epigenetic association as a consequence of its age-related methylation (Li et al., 2019). Furthermore, our docking analysis validates *Rab3b* and *Rab23* as much stronger and more stable interaction partners of *Rab8b* with lowest docking energies that are proportional to the strength of interaction (Figure 6C).

We conversed to the use of rat orthologues in our study to build on FGF2-facilitated *Rab8b* and *Rab31* enrichment to EVs in a more concrete manner by outlining the sub-interactomes only among the close-knitted interactions, though it had restricted us to a limited number of interactions. A further wet-lab validation in brain tissue would directly suffice the specificity and relevance of these interactions. Collectively, this study generates support for the cargo-dependent fate of EVs, because FGF2 triggers enrichment of specific Rabs to EVs and further highlights a Rab-mediated foreground for LB formation and intercellular trafficking defects linked to PD symptoms. Our study supports the idea of allied molecular precursors that could possibly supplement a physiologically favorable state during the onset of PD pathology.

## Data Availability Statement

The EV proteome dataset analyzed in this study can be found in the ProteomeXchange Consortium via the PRIDE partner repository under accession number: PXD014401.

## Ethics Statement

The animal study was reviewed and approved by Local authorities and the animal welfare committee of the Ludwig Maximilian University of Munich, Germany.

## Author Contributions

RK conceived the project. RK, SD, and SM conducted the research. RK, SD, and TK wrote the manuscript. TK, KB, and GH supervised the project. All authors have read and agreed to the published version of the manuscript.

## Conflict of Interest

The authors declare that the research was conducted in the absence of any commercial or financial relationships that could be construed as a potential conflict of interest.

## Footnotes

1 evtrack.org

2 https://string-db.org

3 https://metascape.org/

4 www.mouseproteome.com

5 https://biit.cs.ut.ee/gprofiler/orth

6 www.disgenet.org

7 rgd.mcw.edu

8 https://www.uniprot.org

9 https://swissmodel.expasy.org

10 https://zhanglab.ccmb.med.umich.edu/ModRefiner/

11 https://dokhlab.med.psu.edu/chiron/login.php

12 https://prosa.services.came.sbg.ac.at/prosa.php

13 http://bipspi.cnb.csic.es/xgbPredApp/predictFormFromSeq/

14 http://hdock.phys.hust.edu.cn

15 https://www.r-project.org/
