# Supplementary material for: FGF2 Affects Parkinson’s Disease-Associated Molecular Networks Through Exosomal Rab8b/Rab31
Source: Front Genet. 2020 Sep 25;11:572058. doi: 10.3389/fgene.2020.572058 (PMC7545478; doi:10.3389/fgene.2020.572058)
Supplement: Supplementary file 6 [file Data_Sheet_1.docx]

**Supplementary material**

**Supplementary Figures**

**­­**

**Figure S1. FGF2 increases the abundance of Rab8b and Rab31 in EV-enriched medium pellets.** (A, B) Bar graph and representative Western blot photomicrographs illustrating the protein abundance of *Rab31* in EV-enriched medium pellets from hippocampal neurons. Treatment with bFGF (50 ng/ml; 24 hrs) increases the abundance of *Rab31* (n = 8; P = 0.0011). (C, D) Bar graph and representative Western blot photomicrographs illustrating the protein abundance of *Rab8b* in EV-enriched medium pellets from hippocampal neurons. Treatment with FGF2 (50 ng/ml; 24 hrs) increases the abundance of *Rab31* (n = 8; P = < 0.0001). For quantification of *Rab8b*, the membrane from (B) has been re-reacted with an antibody against *Rab8b* following a mild stripping. Note the close proximity of *Rab31* and *Rab8b* on the Western blot membrane. For statistical comparison an unpaired t-test has been used.

**Figure S2. Exosomal marker expression**. The barplot represents the average log_2_ LFQ intensities from proteome data of exosomal markers *Cd81* (Pvalue:0.0027), *Cd9* (Pvalue:0.000011), *Vamp3* (Pvalue:0), *Rab31* (Pvalue:0.000025), *Rab8b* (Pvalue:0.00043).

**Figure S3.** **Sequence level interaction of Rab proteins.** The barplot denotes interaction score between the *Rab8b-Rab3b* and *Rab8b-Rab23* proteins at sequence level.
